# Cerebral Blood Flow in Predator Stress-Resilient and -Susceptible Rats and Mechanisms of Resilience

**DOI:** 10.3390/ijms232314729

**Published:** 2022-11-25

**Authors:** Marina V. Kondashevskaya, H. Fred Downey, Vadim E. Tseilikman, Valery V. Alexandrin, Kseniya A. Artem’yeva, Valentina V. Aleksankina, Olga B. Tseilikman, Anton A. Pashkov, Anna V. Goryacheva, Irina S. Ivleva, Marina N. Karpenko, Vladislav A. Shatilov, Eugenia B. Manukhina

**Affiliations:** 1 Avtsyn Research Institute of Human Morphology, Petrovsky National Research Center of Surgery, Moscow 117418, Russia; 2 School of Medical Biology, South Ural State University, Chelyabinsk 454080, Russia; 3 Department of Physiology and Anatomy, University of North Texas Health Science Center, Fort Worth, TX 76107, USA; 4 Institute of General Pathology and Pathophysiology, Moscow 125315, Russia; 5 School of Basic Medicine, Chelyabinsk State University, Chelyabinsk 117418, Russia; 6 Pavlov Department of Physiology, Institute of Experimental Medicine, Saint Petersburg 197376, Russia

**Keywords:** predator stress, post-traumatic stress disorder, resilience, cerebral blood flow, endothelial dysfunction, corticosterone, dopamine, hemostasis

## Abstract

Stress-induced conditions are associated with impaired cerebral blood flow (CBF) and increased risk of dementia and stroke. However, these conditions do not develop in resilient humans and animals. Here the effects of predator stress (PS, cat urine scent, ten days) on CBF and mechanisms of CBF regulation were compared in PS-susceptible (PSs) and PS-resilient (PSr) rats. Fourteen days post-stress, the rats were segregated into PSs and PSr groups based on a behavior-related anxiety index (AI). CBF and its endothelium-dependent changes were measured in the parietal cortex by laser Doppler flowmetry. The major findings are: (1) PS susceptibility was associated with reduced basal CBF and endothelial dysfunction. In PSr rats, the basal CBF was higher, and endothelial dysfunction was attenuated. (2) CBF was inversely correlated with the AI of PS-exposed rats. (3) Endothelial dysfunction was associated with a decrease in eNOS mRNA in PSs rats compared to the PSr and control rats. (4) Brain dopamine was reduced in PSs rats and increased in PSr rats. (5) Plasma corticosterone of PSs was reduced compared to PSr and control rats. (6) A hypercoagulation state was present in PSs rats but not in PSr rats. Thus, potential stress resilience mechanisms that are protective for CBF were identified.

## 1. Introduction

Predator stress (PS) can induce post-traumatic stress disorder (PTSD) and other anxiety-like behavior [1,2]. PTSD is a severe psychiatric disorder that develops in individuals after surviving a life-threatening event and may result in both mental and physiological disorders [3,4], especially cardiovascular disease [5,6]. More specifically, PTSD is a recognized risk factor for myocardial infarction and stroke, independent of other cardiovascular risk factors and depression [7,8]. It has been reported that regional cerebral blood flow (CBF) is impaired in patients with PTSD [7,9]. It was suggested [10,11] that this impairment contributes to cognitive disorders observed in patients with PTSD, including a greater risk of dementia [7,12]. Thus, disturbed regulation of CBF can play an important role in the high risk of both cardiovascular and mental disorders following severe stress [7]. Accordingly, special attention should be paid to endothelial dysfunction [13,14,15] as a factor affecting CBF after severe stress.

Not all people exposed to traumatic events develop PTSD [16]. Approximately 60–70% of PTSD-related psychological and physiological disorders do not develop or subside within 1–4 weeks [17]. The long-term response to severe stress apparently depends on individual vulnerability or resilience [17,18,19]. Recently, we compared the effects of PS on the heart of rats segregated into groups susceptible or resilient to experimental PS and showed that following PS, morphological cardiac injury, ECG changes, and impaired exercise tolerance were more pronounced in PS-susceptible (PSs) rats [20]. No such experimental studies of CBF in PS-susceptible and PS-resilient (PSr) animals have been performed, and mechanisms that might account for differences in susceptibility of the cerebrovascular system to the effects of PS have not been investigated.

The present study focuses on the effect of PS on CBF in rats and comparing mechanisms of blood flow regulation in PSr and PSs rats. Our goal is to identify factors that could be implicated in the antistress protection of cerebral circulation and PS resilience. To achieve these goals, rats are first segregated into PSr and PSs groups, based on an anxiety index (AI) determined in an elevated plus maze test [20,21,22,23,24,25,26,27]. Then, basal CBF and endothelial function in these subgroups are compared. For the next step of the study, major mechanisms that could underlie both the CBF protection and the resilience in PSr rats are assessed. These mechanisms include the expression of endothelial NO synthase mRNA and cerebral tissue dopamine, which is protective for CBF and endothelial function due to the stimulation of eNOS expression [28,29]. Also considered is plasma corticosterone, which can provide stress resilience and protection of endothelial function by alleviating the systemic inflammation characteristic of exposure to severe stress [30,31]. Finally, it was found that the PS-resilient phenotype avoids stress-induced hypercoagulation and, hence, is at less risk of cerebrovascular disease, dementia, and stroke.

## 2. Results

Throughout the experiment, all rats were healthy, and the group weights did not significantly differ (*p* > 0.05).

### 2.1. Effect of PS on Behavior in an EPM

The results of the EPM test are shown in Table 1. The AI of control rats (n = 30) was 0.61 ± 0.04. The rats that were exposed to PS and segregated into groups with high AI (AI > 0.8, PSs rats, n = 18) and low AI (AI < 0.8, PSr rats, n = 22). As a result of this segregation, the mean AI of the PSs rats was 51% greater than that of the control rats (*p* < 0.001) and 37% greater than the AI of the PSr rats (*p* < 0.001). The AI of PSr rats was not significantly different from that of the control rats. These data show that, based on anxiety as evaluated by the EPM test, rats exposed to PS clearly expressed two distinctly different phenotypes, stress susceptible or stress resilient. The distribution of these phenotypes was approximately equal (PSs, n = 18/40 and PSr, n = 22/40).

### 2.2. Effect of PS on Cerebral Blood Flow and on Factors That Affect Cerebral Blood Flow

**Figure 1:**
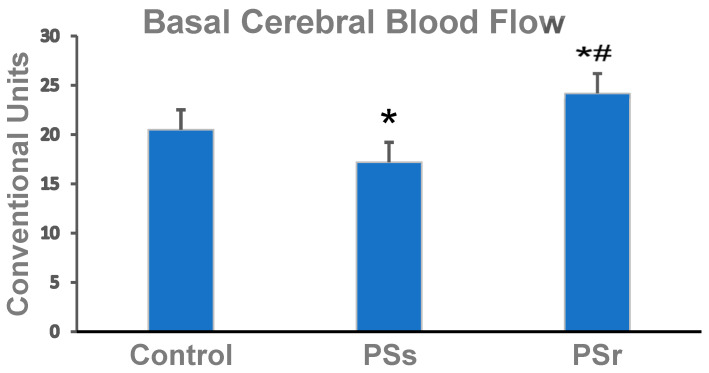
CBF in the parietal cortex of control, PSs, and PSr rats. CBF, cerebral blood flow; PSs, PS-susceptible rats; PSr, PS-resilient rats. * Difference from control, *p* < 0.040; **^#^** Difference from PSs, *p* = 0.043.

The basal CBF in the parietal cortex of PSs rats was 16% less than in the control rats (*p* = 0.038) and 34% less than in PSr (*p* = 0.024) (Figure 1). Interestingly, the basal CBF was 18% greater in PSr rats than in the control rats (*p* = 0.043).

The correlation of AI values with the respective basal CBF values of all rats exposed to PS was computed. The analysis showed that AI was inversely correlated with basal CBF (r = −0.75, *p* = 0.022). Thus, lower anxiety was associated with higher CBF and, therefore, with a better supply of oxygen and nutrients to cerebral structures.

**Figure 2:**
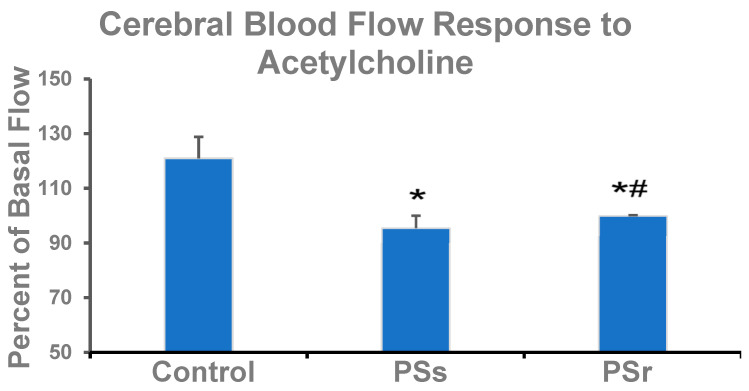
Endothelial function in control, PSs, and PSr rats. Bars show changes in CBF in response to acetylcholine as a percent of basal CBF. CBF, cerebral blood flow; PSs, PS-susceptible rats; PSr, PS-resilient rats. * Difference from the control response, *p* < 0.001. **^#^** Difference from the PSs response, *p* = 0.049.

Ach releases NO from the endothelium, so the response to Ach, as a percentage of basal flow, reflected potential endothelium-dependent vasodilation (EDV) of the cerebral blood vessels (Figure 2). In the control group, Ach increased CBF by 21% (*p* = 0.008 compared to the basal flow). In PSs rats, CBF responded to Ach with a significant decrease, by 5.5%, of the basal flow (*p* < 0.001 compared to the large, positive control response). This inverse response to Ach is typical for endothelial dysfunction.

In PSr rats, there was no significant flow response to Ach (*p* < 0.001 vs. the positive control response), as Ach-stimulated CBF remained unchanged from the basal CBF. The absence of Ach-induced dilation is again consistent with endothelial dysfunction. Still, here the elevated basal CBF of the PSr rats may have dampened the response to Ach. This response to Ach in PSr rats was, however, 4.6% greater than that of PSs rats (*p* = 0.049).

### 2.3. Effect of PS on Cerebral eNOS mRNA

In PSs rats, the content of eNOS mRNA was 86% less (*p*  = 0.010) than in control rats and 85% less in PSr rats (*p*  = 0.016; Table 2). In PSr rats, the eNOS mRNA expression did not differ from the control expression.

### 2.4. Effect of PS on Dopamine in the Cerebral Parietal Cortex

**Figure 3:**
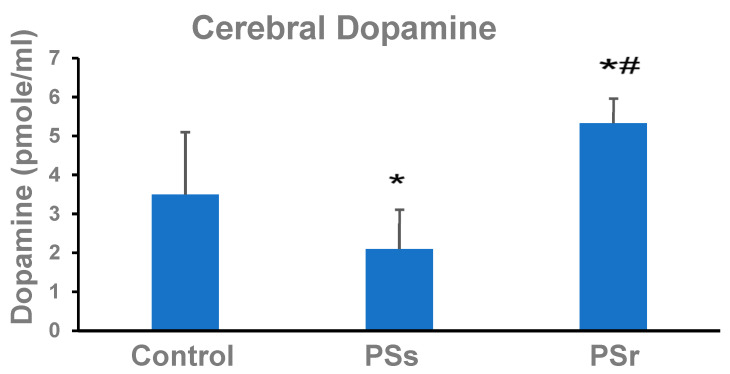
Effect of PS on dopamine concentration in brain parietal cortex of control rats (n = 19), PS-susceptible rats (PSs, n = 9), and PS-resilient rats (PSr, n = 11). * Difference from control, *p* = 0.025. ^#^ Difference from PSs, *p* < 0.001).

DA concentrations in the parietal cortex are shown in Figure 3. In PSs rats, DA was 41% less (*p* = 0.025) than in control rats and 155% less (*p* = 0.004) than in PSr rats. In PSr rats, the DA was 51% greater (*p* < 0.001) than in control rats. These differences in group cerebral dopamine are consistent with the observed differences in group basal cerebral blood flow.

### 2.5. Effect of PS on Plasma Corticosterone

**Figure 4:**
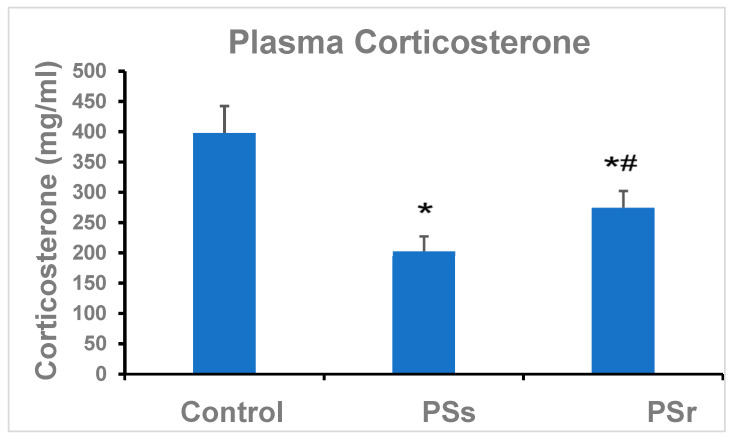
Effect of PS on plasma corticosterone concentration in plasma of control rats (n = 20), PS-susceptible rats (PSs, n = 9), and PS-resilient rats (PSr, n = 11). * Difference from control, *p* < 0.001. ^#^ Difference from PSs, *p* < 0.001).

The effect of PS on the concentration of plasma CORT is shown in Figure 4. In PSs rats, CORT was 49% less (*p* < 0.001) than in control rats and 36% less (*p* > 0.001) than in PSr rats. In PSr rats, CORT was 26% lower (*p* < 0.001) than in control rats.

### 2.6. Effect of PS on Hemostatic Parameters

Regarding the PS-impaired blood coagulation parameters (Table 3), for PSs rats, APTT and PT were 25% and 26% less than in control rats, respectively (*p* < 0.001 for both). The fibrinogen concentration and platelet aggregation were increased by 24% and 28%, respectively (*p* < 0.001 for both).

## 3. Discussion

Psychological stress, anxiety, and PTSD share many common features [32,33]. In this study, we produced stress using the same methods as our previous studies [20,21,22,23,24,25,26,27]. Rats were exposed to repeated PS, and many rats developed anxiety-like behavior by day 14 of the post-stress period. This model has been considered a relevant model of experimental PTSD [1,2], although we now see that only 45% of the rats exposed to PS can be said to develop experimental PTSD. In this study, we used a novel approach that had been used in our recent investigations [20,25,26,27]: rats exposed to PS were segregated into PS-resilient and PS-susceptible groups based on their normal or increased AI, respectively. This approach allowed us to identify and evaluate specific factors contributing to PS resilience or vulnerability.

The major findings of this study are: (1) The susceptibility of rats to PS (PSs rats) was associated with reduced basal CBF in the parietal cortex and with pronounced endothelial dysfunction of cerebral blood vessels. This dysfunction was evident from their paradoxical, inversed response to Ach. For PS-resilient rats (PSr), the basal CBF was higher, and the endothelial dysfunction was less severe than for PSs rats. (2) CBF velocity of rats exposed to PS inversely correlated with their AI values. (3) Endothelial dysfunction of cerebral blood vessels was associated with a significant decrease in eNOS mRNA in PSs rats compared to PSr rats and control rats. (4) The DA concentration in the parietal cortex was reduced in PSs rats and increased in PSr rats. (5) In PSs rats, the blood concentration of CORT was sharply reduced compared to both PSr rats and control rats. (6) The hemostatic parameters of PTs rats indicated a hypercoagulation state, which was not present in PSr rats.

PSs rats had a significantly lower basal CBF than PSr rats, and Ach caused a paradoxical, inverse response of reduced CBF in PS-exposed rats. Together, this is strong evidence of PS-induced endothelial dysfunction in cerebral circulation. Endothelial dysfunction, the impaired endothelium-dependent dilation of blood vessels, is considered to be both a cardiovascular disease marker and a pathogenetic factor contributing to the development and progression of cardio- and cerebrovascular disease, including stroke [34,35]. Endothelial dysfunction is caused by impaired basal and stimulated release and activity of nitric oxide (NO) that functions as a powerful vasodilator, an inhibitor of platelet and leukocyte aggregation, and an anti-inflammatory agent [36]. The contribution of endothelial NO to both maintenance of basal CBF and endothelial-dependent vascular reactivity is traditionally evaluated in both in vivo and in vitro experiments as the dilatory response to Ach, a physiologic stimulator of NO release [36,37]. Importantly, in PSs rats, the endothelial dysfunction was evident as an inverse response to Ach, while in PSr rats, there was no significant response to Ach. In fact, Ach can induce both endothelium-dependent relaxation and endothelium-dependent contraction [38]. The relative strength of these opposing effects determines the resultant relaxation, constriction, or absence of changes [38]. Therefore, the absence of the Ach effect in PSr rats, rather than the inverse reaction, may indicate the preservation of some dilatory potential of the PSr cerebral endothelium, which had a significantly higher basal CBF.

Endothelial dysfunction of cerebral blood vessels associated with PTSD has been reported previously. Studies in middle-aged women, police officers, and male veterans with PTSD showed reduced endothelium-dependent vasodilation [14,39] and biomarkers of endothelial dysfunction [13] correlating with PTSD severity [13,14]. Patients with anxiety and other anxiety disorders also display endothelial dysfunction, evident as impaired flow-mediated vasodilation [40] and decreased numbers of circulating endothelial progenitor cells, which are essential for maintaining normal conditions of the endothelium [41]. Previously, Violanti et al. [42] observed that in persons with more severe PTSD symptoms, the impairment of flow-mediated vasodilation was substantially more pronounced. The current study is the first to demonstrate a negative correlation between CBF and anxiety, i.e., the AI value. Consistently, PSr rats with lower AI values had less pronounced endothelial dysfunction than PSs rats with higher AI values.

In this study, basal CBF was lower and endothelial dysfunction was more pronounced in PSs than in PSr rats, which may reflect differences in eNOS expression or activity. eNOS provides both basal and stimulated NO production. Basal endothelial NO production by eNOS regulates cerebral vascular tone and maintains resting CBF [37]. CBF attenuation is associated with the downregulation of eNOS expression [43]. By normalizing CBF, eNOS protects the brain from various pathological conditions due to microvascular abnormalities, such as cerebral ischemia, hypertension, and brain hypoperfusion. The ability of eNOS to produce adequate amounts of available NO can be assessed by the endothelium-dependent vasorelaxation to Ach [37], as was performed in this study.

Acute and chronic stresses are known to cause dysregulation of eNOS through increased oxidative stress, inflammation, and increased glucocorticoid levels [44]. In monkeys, chronic mental stress-induced endothelial injury is mediated by β1-adrenoceptor activation [45]. Chronic immobilization stress (120 min/day, 14 days) of rats decreased the arterial eNOS mRNA due to vascular oxidative stress [46]. Under stressful conditions, glucocorticoids are the major agents that downregulate the transcription and activity of NOS via a feedback mechanism [47,48]. Liu et al. [49] reported that cortisol decreased the expression of eNOS in human endothelial cells. Consistent results were obtained on rats administered with dexamethasone [50]; dexamethasone increased blood pressure, caused endothelial dysfunction, and downregulated the expression of eNOS. In the present study, we showed for the first time that a greater PS-induced decrease in eNOS expression was associated with more pronounced impairments of basal CBF and endothelial function in PSs rats. Earlier, we showed that oxidative stress and inflammatory cytokines were higher in rats vulnerable to experimental PTSD compared to resilient rats [20]. Apparently, these factors contributed to more severe damage to eNOS in PSs rats.

Changes in CBF in the parietal cortex were consistent with changes in DA concentrations in the same brain area. Thus, protection of the cerebral circulation was associated with increased DA concentrations. In previous studies, DA has been related to PTSD resilience [51]. Low DA is generally consistent with an increased risk for PTSD [52], and this link is genetically predetermined [53]. In clinical studies, a so-called “pro-dopamine regulator,” nutraceutical KB220Z, was successfully used to treat PTSD-related recurrent distressing nightmares [54,55]. In our previous PS study [24], cerebral DA concentrations were also decreased in PS-exposed rats. Here, for the first time, we show a post-stress increase in cerebral DA in rats resilient to PS.

In chronic stress, decreased DA in the brain is associated with local activation and degeneration of the microglia, while administration of exogenous DA maintains autoregulation of damaged structures and prevents their necrosis [56]. DA protects cerebral autoregulation and prevents hippocampal necrosis after traumatic brain injury via a block of ERK MAPK in juvenile pigs. Also, DA is involved in the regulation of microcirculation and acts as a direct vasodilator [57,58].

One of the mechanisms underlying the protective effects of DA is its direct effect on endothelial function. DA was shown to reverse the spasms of isolated porcine arteries induced by cerebrospinal fluid from patients with cerebral vasospasms after a subarachnoid hemorrhage [28]. This DA effect was due to the stimulation of the expression of endothelial NO synthase (eNOS) mediated by D2 receptors. Haloperidol, a D2 receptor antagonist, prevented this effect. Wang et al. [59] reported that the protective effect of DA against endothelial dysfunction was also mediated through D4 receptors. Clinical and experimental studies showed that DA could directly increase basal local CBF, and this increase is blocked by haloperidol [29,60,61]. Thus, the increased DA improves cerebral microcirculation and alleviates anxiety, which facilitates the development of PS resilience.

Analyzing cerebral DA using homogenized brain tissue is a limitation of this study since this method cannot distinguish the differences between intracellular and extracellular DA content. In future experiments, pharmacological methods using agonists or antagonists should also be performed.

Lowered plasma cortisol is observed in patients with PTSD [62]. In our study, plasma CORT concentrations were decreased in both PSs and PSr rats. However, this decrease was significantly more pronounced in PSs than in PSr rats. This finding is consistent with our previous results showing that in low-anxiety rats with experimental PTSD/anxiety-like behavior, the reduction in the plasma CORT concentration was transient, while the high-anxiety rats demonstrated sustained CORT reduction [26]. Also, in other studies [63], PTSD-susceptible rats had decreased plasma concentrations of CORT as well as reduced corticotropin-releasing factor expression.

Glucocorticoids are essential for alleviating systemic inflammation associated with PTSD due to the suppression of cytokine secretion [30,31]. The close association of PTSD with systemic inflammation and oxidative stress is confirmed by both clinical and experimental studies [64,65,66,67]. The balance between pro- and anti-inflammatory cytokine response is considered as a marker for PTSD resilience [64,68]. Previously we showed that the pro- and anti-inflammatory cytokine balance was an important determinant of rat heart resilience or injury in experimental PTSD [20]. In the brain, neuroinflammation induced by mental stress exposure causes the excessive formation of reactive oxygen species, which, in turn, induces the uncoupling and dysfunction of vascular eNOS and reduces NO availability [44]. Patients with PTSD have lower plasma cortisol levels [69,70], which was suggested to contribute to the inflammatory activation of endothelial cells [71,72,73]. Thus, the different decrease in plasma CORT may be one of the mechanisms for the less pronounced endothelial dysfunction in PSr rats compared to PSs.

The evaluation of hemostatic parameters showed that stress resilience and the ensuing improvement of endothelial function might provide protection against cerebrovascular disease and stroke. PTSD is associated with premature death caused by thromboembolism [74,75]. Our study showed that in PSr rats, hemostatic parameters did not differ from those of control rats, whereas in PSs rats, APTT and PT were decreased, and fibrinogen concentration and platelet aggregation were increased. These results are consistent with clinical reports that both acute and chronic stress, including PTSD, are associated with procoagulation disorders and predisposition to thrombogenesis [76]. Thus, PTSD caused hypercoagulation associated with increased concentrations of factor VIII, von Willebrand factor, and fibrinogen and with platelet aggregation. Also, increased PT and decreased APTT were observed in people exposed to chronic stress [77,78]. Furthermore, PTSD severity was shown to correlate positively with plasma levels of the procoagulants, factor VIII and fibrinogen [79].

One important mechanism of stress-induced hypercoagulation is endothelial dysfunction. Normally, the endothelium provides a balance between coagulation and fibrinolysis by producing thrombin synthesis and activity inhibitors. However, damaged endothelium with insufficient production or availability of NO loses its anticoagulant and profibrinolytic properties of endothelial cells, resulting in exaggerated hypercoagulability during acute stress [80,81]. Indeed, in our study, we showed that PSs rats that were characterized with a procoagulation phenotype also had a more pronounced endothelial dysfunction and impaired eNOS mRNA expression. This was distinct from PSr rats, whose coagulation parameters, fibrinogen concentration, and platelet activation did not significantly differ from the control.

One limitation of this study is the use of male animals only. There are many reports of sex differences in the incidence of anxiety disorders, showing that women are twice as vulnerable as men to PTSD and that their symptoms and PTSD-related comorbidities are different [82,83,84]. However, despite the obvious importance of experimental data from females, such studies are scarce; most experiments have been performed on males. Clearly, this investigation must be extended to include females. Also, some aspects of our discussion are speculative and require further experimentation for their validation.

In summary, this study showed, for the first time, that higher stress susceptibility of rats is associated with cerebrovascular disorders, evident as reduced basal cerebral blood flow and endothelial dysfunction. These findings suggest several potential mechanisms that could underlie the stress vulnerability of cerebral blood vessels, including reduced levels of endothelial NO synthase, DA, and CORT, as well as a state of hypercoagulation. All these mechanisms remained practically undisturbed in stress-resilient rats, thus providing protection against detrimental stress factors, such as inflammation and oxidative stress.

## 4. Material and Methods

### 4.1. Animals and Experimental Procedure

The study was performed on 70 sexually mature, healthy male Wistar rats weighing 180–200 g. The rats were kept in standard vivarium conditions (controlled temperature, 22–25 °C; humidity, 55%), 10 rats per cage. A 12:12 h light–dark cycle was maintained with lights on between 7 AM and 7 PM. The animals had ad libitum access to water and pelleted food, a standard laboratory diet. The rats were randomly assigned to two groups: control rats (n = 30) and rats exposed to PS (n = 40). All experimental procedures were conducted according to the guidelines of the Declaration of Helsinki and approved by the Institutional Ethics Committee of the Avtsyn Research Institute of Human Morphology (protocol #26 of 13 September 2021).

### 4.2. Exposure to PS

A modified PS model was used, initially described by Cohen and Zohar [2] and as used in our prior studies [20,21,22,23,24,25,26,27]. PS was induced by exposing rats to cat urine scent for 10 min daily for 10 days. PS rats were then given 14 days of rest under stress-free conditions. Control rats were rested stress-free during this 24-day period.

### 4.3. Behavioral Testing

The PS outcome was evaluated with an elevated plus maze (EPM) test, as employed in our prior studies [20,21,22,23,24,25,26,27]. This test was performed on the PS and control rats following the rest periods described above. The total duration of the test was 10 min. Control and PS rats were tested together in a blind fashion. The behavior of rats in the EPM was recorded and tracked using the SMART video system and analyzed with SMART 3.0 software. The number of entries into the open and closed arms of the EPM and the time spent in the open and closed arms were recorded. Based on these measurements, the AI was calculated [85]:

AI = 1 − {[(time in open arms/Σ time on maze) + (number of entries into open arms/Σ number of all entries)]/2}.

An AI > 0.8 was considered a marker for the presence of anxiety-like behavior; rats with AI > 0.8 were assigned to the PS-susceptible group (PSs group), and rats with AI < 0.8 were assigned to the PS-resilient group (PSr group). The AI discriminant of 0.8 was set based on the AI of naive rats measured in preliminary experiments for this study and on historical AIs of control rats as reported earlier [20,26,27].

### 4.4. Measurement of Regional CBF

The rats were anesthetized with chloral hydrate (400 mg/kg) and placed in a stereotaxic instrument. A hole was drilled in the parietal region to gain access to the parietal cortex. The local CBF was continuously recorded with an ALF-21 laser doppler flowmeter (Transonic Systems, Inc, Ithaca, NY, USA). The recording was performed in PT1: 5 mm from bregma and 3 mm from the midline. After the stabilization of the local CBF, the basal CBF was recorded. Then the endothelium-dependent response of cerebral vessels was elicited by the injection of 10^−5^ M acetylcholine chloride (Ach) into the carotid artery and assessed as the change in CBF expressed as a percentage of the basal CBF.

### 4.5. Blood and Tissue Collection and Storage

On the day after the EPM test, rats were sacrificed by decapitation under diethyl ether anesthesia, and samples of trunk blood and parietal brain cortex were collected.

For the measurement of corticosterone (CORT), blood was transferred to sterile glass tubes containing K_2_EDTA and centrifuged at 4 °C and 3000× *g* for 10 min. Plasma was separated into aliquots, which were frozen at −80 °C.

For measurement of hemostasis parameters, blood was collected into a 3.8% sodium citrate solution (9:1, V/V). These samples were centrifuged immediately at 160× *g* for 15 min at room temperature to prepare platelet-rich plasma (PRP) for measuring platelet aggregation. PRP was transferred into plastic tubes, and the remaining blood was centrifuged at 3000× *g* for 10 min to obtain platelet-poor plasma (PPP) for measuring coagulation parameters.

The parietal cortex (PT1, according to Paxinos and Watson’s 2014 atlas) was located, dissected from the fresh brains, cooled on ice, and then frozen in liquid nitrogen.

### 4.6. Measurement of eNOS mRNA

#### 4.6.1. RNA Isolation

The total RNA was isolated from brain tissue with TRIzol Reagent (Invitrogen, Oxford, UK). The RNA concentration was measured using a NanoDrop 2000 spectrophotometer (Thermo Scientific, Waltham, MA, USA) following standard procedures. The purity of RNA samples was verified by confirming that each had an optical density ratio of A260/A280 > 1.8. To verify the integrity of the samples, the 18S/28S RNA ratio was analyzed after electrophoresis in 1.4% agarose gel.

#### 4.6.2. cDNA Synthesis and Real-Time RT–PCR

Two μg of total RNA was used for cDNA synthesis using high-capacity cDNA reverse transcription kits (Applied Biosystems, Singapore). Quantitative RT–PCR was performed using Evrogen 5x qPCR mix–HS SYBR. Primers were designed with the Primer-BLAST software (NCBI, Bethesda, MD, USA); the primer sequences are presented in Table 4. The PCR parameters were as follows: initial denaturation (one cycle at 95 °C for 15 min); 40 cycles of denaturation, amplification, and quantification (95 °C for 15 s, annealing temperature for 30 s, and 72 °C for 5 s); and the melting curve (starting at 65 °C and gradually increasing to 95 °C). Cycr mRNA was used as an internal control. The ΔΔCt method was used to determine the fold increase of genes relative to the control group. Each value was combined from two independent PCR replicates for each cDNA sample, obtained from five animals.

### 4.7. Dopamine Measurement

Samples of parietal cortex (PT1 according to Paxinos and Watson’s 2014 atlas) were homogenized in 0.1 M perchloric acid with added 3,4 dihydroxybenzylamine (0.5 nmol/L) as the internal standard. After homogenization, the samples were centrifuged (10,000× *g* for 10 min at 4 °C), and the supernatants were withdrawn. High-performance liquid chromatography analysis with electrochemical detection was performed on an LC 304T (BAS, West Lafayette, IN, USA) with a Rheodyne 7125 injector. Supernatant (20 µL) was injected into a thermostatic (25C) Ultrasphere C18 analytical column. The mobile phase consisted of a 0.1 M phosphate-citrate buffer containing 0.3 mM sodium octanesulphonate, 0.1 mM EDTA, and 8% (*v*/*v*) acetonitrile (pH 3.2). Electrochemical detection was performed with a glassy carbon electrode at +0.85 potential vs. Ag/AgCI reference electrode. The mobile phase velocity was 0.7 mL/min. Standards were prepared in 0.1 M HCIO_4_ at 100 µg/mL supplemented with 0.2 mM sodium metabisulfite as a preservative. Working standards were prepared daily. The final concentration of dopamine in the tissue sample was expressed as pg/mg of tissue, using an external calibration curve, as previously described [24].

### 4.8. Corticosterone Measurement

Plasma corticosterone was measured in duplicate with a multiplate ANTHOS 2010 (Austria) analyzer and commercial radioimmunoassay kits (Cort, IBL, Hamburg, Germany) according to the manufacturer’s instructions.

### 4.9. Measurement of Hemostatic Parameters

Platelet aggregation was measured in PRP. After the addition of ADP (5 μM), platelet aggregation was measured using a platelet aggregometer (SC-2000, Saikexide Instrument Co. Ltd., Hong Kong, China).

PPP was used to measure the coagulation parameters, including prothrombin time (PT), activated partial thromboplastin time (APTT), and fibrinogen time (FT), using commercial kits according to the manufacturer’s instructions and a Thrombostat 2 coagulometer (Behnk Elektronik, Norderstedt, Germany).

### 4.10. Statistical Analysis

Data were analyzed with SPSS 24 (IBM, New York, NY, USA), STATISTICA 10.0 (StatSoft, Tulsa, OK, USA), Rstudio (RStudio, Boston, MA, USA) and Excel (Microsoft, Redmond, WA, USA) software. The normality of data distributions was confirmed with the Shapiro–Wilk test. These data were analyzed with a parametric, one-factor ANOVA followed by Fisher’s post hoc tests. Data are presented as the mean ± standard deviation (SD). Relationships between variables were examined by Spearman correlation analysis. A value of *p* < 0.05 was considered significant.

## 5. Conclusions

Using rats segregated into the groups ‘susceptible’ and ‘resilient to PS’, this study showed that susceptible rats with pronounced anxiety-like behavior had reduced basal cerebral blood flow and endothelial dysfunction of the cerebral blood vessels. We identified major factors implicated in both the maintenance of endothelial function and general stress resilience and compared them in PSr and PSs rats. These factors, including the expression of eNOS mRNA, brain concentration of DA, and plasma concentration of CORT, were maintained close to control levels in PSr rats but were disturbed in PSs rats. Preservation of normal DA and CORT would provide adequate protection against the detrimental effects of stress-induced inflammation and oxidative stress on the endothelium. Evaluation of hemostatic parameters suggested that the impaired endothelial function and NO production in cerebral blood vessels can mediate hypercoagulation and formation of a prothrombotic, hemocoagulation state, which would contribute to an increased risk of cerebrovascular disease and stroke. Further study of the mechanisms of traumatic stress resilience will help in developing new approaches for the prevention of post-stress disease.

## Figures and Tables

**Table 1:** Behavioral data from elevated plus maze test.

Variable	Control Rats (n = 30)	PSs Rats (n = 18)	PSr Rats (n = 22)
Number of entries into closed arms	10.3 ± 0.6	6.1 ± 0.3 *	9.4 ± 0.5 ^#^
Number of entries into open arms	6.3 ± 0.4	0.9 ± 0.1 *	5.5 ± 0.3 ^#^
Time spent in closed arms, s	346 ± 20	589 ± 32 *	431± 26 ^#^
Time spent in open arms, s	255 ± 12	10.2 ± 0.6 *	170 ± 10 ^#^
Anxiety index	0.61 ± 0.04	0.92 ± 0.05 *	0.67 ± 0.04 ^#^

Data are mean ± SD. PSs, PS-susceptible rats; PSr, PS-resilient rats. * Difference from control, *p* < 0.001. ^#^ Difference from PSs, *p* < 0.001.

**Table 2:** Effect of PS on eNOS mRNA expression in control, PSs, and PSs rats.

Groups	mRNA eNOS
Control (n = 5)	3.44 ± 2.63
PSs (n = 5)	0.5 ± 0.01 *
PSr (n = 10)	3.3 ± 0.25 ^#^

Data are eNOS mRNA content in relative units (eNOS/GAPDH). PSs, susceptible rats; PSr, PS-resilient rats; eNOS, endothelial NO-synthase. * Difference from control, *p* = 0.01; ^#^ Difference from PSs, *p* < 0.02.

**Table 3:** Data from blood coagulation tests.

Variable	Control Rats (n = 10)	PSs Rats (n = 10)	PSr Rats (n = 10)
APTT, s	23.6 ± 1.4	17.3 ± 0.7 *^,#^	22.9 ± 1.2
PT, s	13.9 ± 0.7	10.1 ± 0.6 *^,#^	13.6 ± 0.6
Fibrinogen concentration, g/L	2.7 ± 0.1	3.6 ± 0.4 *^,#^	2.8 ± 0.2
Platelet aggregation, %	48 ± 2	69 ± 2 *^,#^	50 ± 3

Data are mean ± SD. PSs, PS-susceptible rats; PSr, PS-resilient rats; APTT, activated partial thromboplastin time; PT, prothrombin time; FG, fibrinogen, PA, platelet aggregation. * Difference from control, *p* < 0.001; ^#^ Difference from PSr, *p* < 0.001).

**Table 4:** The primer sequences.

Name of the Gene	Primer Sequence 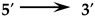	Annealing Temperature, °C
eNOS	F GATCCTAACTTGCCTTGCATCCT R TGTAATCGGTCTTGCCAGAATCC	58

## Data Availability

Not applicable.

## References

[1] Cohen H., Kozlovsky N., Alona C., Matar M.A., Joseph Z. (2012). Animal model for PTSD: From clinical concept to translational research. Neuropharmacology.

[2] Cohen H., Zohar J. (2004). Animal models of post-traumatic stress disorder: The use of cut off behavioral criteria. Ann. N. Y. Acad. Sci..

[3] Gupta M.A. (2013). Review of somatic symptoms in post-traumatic stress disorder. Int. Rev. Psychiatry.

[4] Pietrzak R.H., Goldstein R.B., Southwick S.M., Grant B.F. (2011). Medical comorbidity of full and partial posttraumatic stress disorder in US adults: Results from Wave 2 of the National Epidemiologic Survey on Alcohol and Related Conditions. Psychosom. Med..

[5] Edmondson D., Kronish I.M., Shaffer J.A., Falzon L., Burg M.M. (2013). Posttraumatic stress disorder and risk for coronary heart disease: A meta-analytic review. Am. Heart J..

[6] Seligowski A.V., Webber T.K., Marvar P.J., Ressler K.J., Philip N.S. (2022). Involvement of the brain-heart axis in the link between PTSD and cardiovascular disease. Depress. Anxiety.

[7] Ogoh S. (2017). Relationship between cognitive function and regulation of cerebral blood flow. J. Physiol. Sci..

[8] Remch M., Laskaris Z., Flory J., Mora-McLaughlin C., Morabia A. (2018). Post-traumatic stress disorder and cardiovascular diseases: A cohort study of men and women involved in cleaning the debris of the World Trade Center Complex. Circ. Cardiovasc. Qual. Outcomes..

[9] Liu Y., Li B., Feng N., Pu H., Zhang X., Lu H., Yin H. (2016). Perfusion deficits and functional connectivity alterations in memory-related regions of patients with post-traumatic stress disorder. PLoS ONE.

[10] Ogoh S., Yoo J.K., Badrov M.B., Parker R.S., Anderson E.H., Wiblin J.L., North C.S., Suris A., Fu Q. (2018). Cerebral blood flow regulation and cognitive function in women with posttraumatic stress disorder. J. Appl. Physiol..

[11] Scott J.C., Matt G.E., Wrocklage K.M., Crnich C., Jordan J., Southwick S.M., Krystal J.H., Schweinsburg B.C. (2015). A quantitative meta-analysis of neurocognitive functioning in posttraumatic stress disorder. Psychol. Bull..

[12] Elias A., Rowe C., Hopwood M. (2021). Risk of dementia in posttraumatic stress disorder. J. Geriatr. Psychiatry Neurol..

[13] Von Känel R., Hepp U., Traber R., Kraemer B., Mica L., Keel M., Mausbach B.T., Schnyder U. (2008). Measures of endothelial dysfunction in plasma of patients with posttraumatic stress disorder. Psychiatry Res..

[14] Grenon S.M., Owens C.D., Alley H., Perez S., Whooley M.A., Neylan T.C., Aschbacher K., Gasper W.J., Hilton J.F., Cohen B.E. (2016). Posttraumatic stress disorder is associated with worse endothelial function among veterans. J. Am. Heart Assoc..

[15] Cleveland S., Reed K., Thomas J.L., Ajijola O.A., Ebrahimi R., Hsia T., Lazarov A., Montoya A.K., Neria Y., Shimbo D. (2021). Key dimensions of post-traumatic stress disorder and endothelial dysfunction: A protocol for a mechanism-focused cohort study. BMJ Open.

[16] Breslau N., Chilcoat H.D., Kessler R.C., Davis G.C. (1999). Previous exposure to trauma and PTSD effects of subsequent trauma: Results from the Detroit Area Survey of Trauma. Am. J. Psychiatry.

[17] Horn S.R., Charney D.S., Feder A. (2016). Understanding resilience: New approaches for preventing and treating PTSD. Exp. Neurol..

[18] Osorio C., Probert T., Jones E., Young A.H., Robbins I. (2017). Adapting to stress: Understanding the neurobiology of resilience. Behav. Med..

[19] Ryan M., Ryznar R. (2022). The molecular basis of resilience: A narrative review. Front. Psychiatry.

[20] Manukhina E.B., Tseilikman V.E., Komelkova M.V., Lapshin M.S., Goryacheva A.V., Kondashevskaya M.V., Mkhitarov V.A., Lazuko S.S., Tseilikman O.B., Sarapultsev A.P. (2021). Cardiac injury in rats with experimental posttraumatic stress disorder and mechanisms of its limitation in experimental posttraumatic stress disorder-resistant rats. J. Appl. Physiol. (1985).

[21] Lazuko S.S., Kuzhel O.P., Belyaeva L.E., Manukhina E.B., Downey H.F., Tseilikman O.B., Komelkova M.V., Tseilikman V.E. (2018). Posttraumatic stress disorder disturbs coronary tone and its regulatory mechanisms. Cell Mol. Neurobiol..

[22] Manukhina E.B., Tseilikman V.E., Tseilikman O.B., Komelkova M.V., Kondashevskaya M.V., Goryacheva A.V., Lapshin M.S., Platkovskii P.O., Alliluev A.V., Downey H.F. (2018). Intermittent hypoxia improves behavioral and adrenal gland dysfunction induced by post-traumatic stress disorder in rats. J. Appl. Physiol..

[23] Manukhina E.B., Tseilikman V.E., Karpenko M.N., Pestereva N.S., Tseilikman O.B., Komelkova M.V., Kondashevskaya M.V., Goryacheva A.V., Lapshin M.S., Platkovskii P.O. (2020). Intermittent hypoxic conditioning alleviates post-traumatic stress disorder-induced damage and dysfunction of rat visceral organs and brain. Int. J. Mol. Sci..

[24] Dremencov E., Lapshin M., Komelkova M., Alliluev A., Tseilikman O., Karpenko M., Pestereva N., Manukhina E., Downey H.F., Tseilikman V. (2019). Chronic predator scent stress alters serotonin and dopamine levels in the rat thalamus and hypothalamus, respectively. Gen. Physiol. Biophys..

[25] Tseilikman V., Dremencov E., Maslennikova E., Ishmatova A., Manukhina E., Downey H.F., Klebanov I., Tseilikman O., Komelkova M., Lapshin M.S. (2019). Post-traumatic stress disorder chronification via monoaminoxidase and cortisol metabolism. Horm. Metab. Res..

[26] Tseilikman V., Komelkova M., Kondashevskaya M.V., Manukhina E., Downey H.F., Chereshnev V., Chereshneva M., Platkovskii P., Goryacheva A., Pashkov A. (2021). A rat model of post-traumatic stress syndrome causes phenotype-associated morphological changes and hypofunction of the adrenal gland. Int. J. Mol. Sci..

[27] Tseilikman V., Lapshin M., Klebanov I., Chrousos G., Vasilieva M., Pashkov A., Fedotova J., Tseilikman D., Shatilov V., Manukhina E. (2022). The link between activities of hepatic 11beta-hydroxysteroid dehydrogenase-1 and monoamine oxidase-A in the brain following repeated predator stress: Focus on heightened anxiety. Int. J. Mol. Sci..

[28] Pyne-Geithman G.J., Caudell D.N., Cooper M., Clark J.F., Shutter L.A. (2009). Dopamine D2-receptor-mediated increase in vascular and endothelial NOS activity ameliorates cerebral vasospasm after subarachnoid hemorrhage in vitro. Neurocrit. Care.

[29] Chen Y., Pressman P., Simuni T., Parrish T.B., Gitelman D.R. (2015). Effects of acute levodopa challenge on resting cerebral blood flow in Parkinson’s Disease patients assessed using pseudo-continuous arterial spin labeling. Peer J..

[30] Sapolsky R.M., Romero L.M., Munck A.U. (2000). How do glucocorticoids influence stress responses? Integrating permissive, suppressive, stimulatory, and preparative actions. Endocr. Rev..

[31] Baker D.G., Ekhator N.N., Kasckow J.W., Hill K.K., Zoumakis E., Dashevsky B.A., Chrousos G.P., Geracioti T.D. (2001). Plasma and cerebrospinal fluid Interleukin-6 concentrations in posttraumatic stress disorder. Neuroimmunomodulation.

[32] De Berardis D., Vellante F., Fornaro M., Anastasia A., Olivieri L., Rapini G., Serroni N., Orsolini L., Valchera A., Carano A. (2020). Alexithymia, suicide ideation, affective temperaments and homocysteine levels in drug naïve patients with post-traumatic stress disorder: An exploratory study in the everyday ‘real world’ clinical practice. Int. J. Psychiatry Clin. Pract..

[33] Smagin D.A., Kovalenko I.L., Galyamina A.G., Belozertseva I.V., Tamkovich N.V., Baranov K.O., Kudryavtseva N.N. (2021). Chronic lithium treatment affects anxious behaviors and the expression of serotonergic genes in midbrain raphe nuclei of defeated male mice. Biomedicines.

[34] Cosentino F., Rubattu S., Savoia C., Venturelli V., Pagannonne E., Volpe M. (2001). Endothelial dysfunction and stroke. J. Cardiovasc. Pharmacol..

[35] Sfera A., Osorio C., Rahman L., Zapata-Martín Del Campo C.M., Maldonado J.C., Jafri N., Cummings M.A., Maurer S., Kozlakidis Z. (2021). PTSD as an endothelial disease: Insights from COVID-19. Front. Cell Neurosci..

[36] Benincasa G., Coscioni E., Napoli C. (2022). Cardiovascular risk factors and molecular routes underlying endothelial dysfunction: Novel opportunities for primary prevention. Biochem. Pharmacol..

[37] Atochin D.N., Huang P.L. (2011). Role of endothelial nitric oxide in cerebrovascular regulation. Curr. Pharm. Biotechnol..

[38] Rosenblum W.I. (2018). Endothelium-dependent responses in the microcirculation observed in vivo. Acta Physiol..

[39] Thurston R.C., Barinas-Mitchell E., von Känel R., Chang Y., Koenen K.C., Matthews K.A. (2018). Trauma exposure and endothelial function among midlife women. Menopause.

[40] Celano C.M., Daunis D.J., Lokko H.N., Campbell K.A., Huffman J.C. (2016). Anxiety disorders and cardiovascular disease. Curr. Psychiatry Rep..

[41] Felice F., Di Stefano R., Pini S., Mazzotta G., Bovenzi F.M., Bertoli D., Abelli M., Borelli L., Cardini A., Lari L. (2015). Influence of depression and anxiety on circulating endothelial progenitor cells in patients with acute coronary syndromes. Hum. Psychopharmacol..

[42] Violanti J.M., Andrew M., Burchfiel C.M., Hartley T.A., Charles L.R., Miller D.B. (2007). Post-traumatic stress symptoms and cortisol patterns among police officers. Policing An Intl. J. Police Strateg. Mgmt..

[43] Jesmin S., Togashi H., Mowa C.N., Ueno K., Yamaguchi T., Shibayama A., Miyauchi T., Sakuma I., Yoshioka M. (2004). Characterization of regional cerebral blood flow and expression of angiogenic growth factors in the frontal cortex of juvenile male SHRSP and SHR. Brain Res..

[44] Daiber A., Kröller-Schön S., Oelze M., Hahad O., Li H., Schulz R., Steven S., Münzel T. (2020). Oxidative stress and inflammation contribute to traffic noise-induced vascular and cerebral dysfunction via uncoupling of nitric oxide synthases. Redox Biol..

[45] Skantze H.B., Kaplan J., Pettersson K., Manuck S., Blomqvist N., Kyes R., Williams K., Bondjers G. (1998). Psychosocial stress causes endothelial injury in cynomolgus monkeys via β1- adrenoceptor activation. Atherosclerosis.

[46] Chung I.M., Kim Y.M., Yoo M.H., Shin M.K., Kim C.K., Suh S.H. (2010). Immobilization stress induces endothelial dysfunction by oxidative stress via the activation of angiotensin II/its type I receptor pathway. Atherosclerosis.

[47] López-Figueroa M.O., Day H.E., Akil H., Watson S.J. (1998). Nitric oxide in the stress axis. Histol. Histopath..

[48] Toda N., Nakanishi-Toda M. (2011). How mental stress affects endothelial function. Pflugers Arch..

[49] Liu Y., Mladinov D., Pietrusz J.L., Usa K., Liang M. (2009). Glucocorticoid response elements and 11 β-hydroxysteroid dehydrogenases in the regulation of endothelial nitric oxide synthase expression. Cardiovasc. Res..

[50] Wallerath T., Whited K., Schäfer S.C., Schwarz P.M., Wohlfart P., Kleinert H., Lehr H.A., Lemmer B., Förstermann U. (1999). Down–regulation of the expression of endothelial NO synthase is likely to contribute to glucocorticoid-mediated hypertension. Proc. Natl. Acad. Sci. USA.

[51] Blum K., Gondré-Lewis M.C., Modestino E.J., Lott L., Baron D., Siwicki D., McLaughlin T., Howeedy A., Krengel M.H., Oscar-Berman M. (2019). Understanding the scientific basis of post-traumatic stress disorder (PTSD): Precision behavioral management overrides stigmatization. Mol. Neurobiol..

[52] Blum K., Giordano J., Oscar-Berman M., Bowirrat A., Simpatico T., Barh D. (2012). Diagnosis and healing in veterans suspected of suffering from post-traumatic stress disorder (PTSD) using reward gene testing and reward circuitry natural dopaminergic activation. J. Genet. Syndr. Gene Ther..

[53] Roy-Byrne P., Arguelles L., Vitek M.E., Goldberg J., Keane T.M., True W.R., Pitman R.K. (2004). Persistence and change of PTSD symptomatology—A longitudinal co-twin control analysis of the Vietnam Era Twin Registry. Soc. Psychiatry Psychiatr. Epidemiol..

[54] McLaughlin T., Blum K., Oscar-Berman M., Febo M., Agan G., Fratantonio J.L., Simpatico T., Gold M.S. (2015). Putative dopamine agonist (KB220Z) attenuates lucid nightmares in PTSD patients: Role of enhanced brain reward functional connectivity and homeostasis redeeming joy. J. Behav. Addict..

[55] McLaughlin T., Blum K., Oscar-Berman M., Febo M., Demetrovics Z., Agan G., Fratantonio J., Gold M.S. (2015). Using the neuroadaptagen KB200z to ameliorate terrifying, lucid nightmares in RDS patients: The role of enhanced, brain-reward, functional connectivity and dopaminergic homeostasis. J. Reward. Defic. Syndr..

[56] Curvello V., Hekierski H., Pastor P., Vavilala M.S., Armstead W.M. (2017). Dopamine protects cerebral autoregulation and prevents hippocampal necrosis after traumatic brain injury via block of ERK MAPK in juvenile pigs. Brain Res..

[57] Afonso-Oramas D., Cruz-Muros I., Castro-Hernández J., Salas-Hernández J., Barroso-Chinea P., García-Hernández S., Lanciego J.L., González-Hernández T. (2014). Striatal vessels receive phosphorylated tyrosine hydroxylase-rich innervation from midbrain dopaminergic neurons. Front. Neuroanat..

[58] Melamed E., Lavy S., Cooper G., Bentin S. (1978). Regional cerebral blood flow in parkinsonism. Measurement before and after levodopa. J. Neurol. Sci..

[59] Wang H., Yao Y., Liu J., Cao Y., Si C., Zheng R., Zeng C., Guan H., Li L. (2019). Dopamine D_4_ receptor protected against hyperglycemia-induced endothelial dysfunction via PI3K/eNOS pathway. Biochem. Biophys. Res. Commun..

[60] von Essen C., Zervas N.T., Brown D.R., Koltun W.A., Pickren K.S. (1980). Local cerebral blood flow in the dog during intravenous infusion of dopamine. Surg. Neurol..

[61] Martens M., McConnell F.K., Filippini N., Mackay C.E., Harrison P.J., Tunbridge E.M. (2021). Dopaminergic modulation of regional cerebral blood flow: An arterial spin labelling study of genetic and pharmacological manipulation of COMT activity. Neuroimage.

[62] Yehuda R. (2002). Post-traumatic stress disorder. N. Engl. J. Med..

[63] Skórzewska A., Lehner M., Wisłowska-Stanek A., Turzyńska D., Sobolewska A., Krząścik P., Szyndler J., Maciejak P., Chmielewska N., Kołosowska K. (2020). Individual susceptibility or resistance to posttraumatic stress disorder-like behaviours. Behav. Brain Res..

[64] Miller M.W., Lin A.P., Wolf E.J., Miller D.R. (2018). Oxidative stress, inflammation, and neuroprogression in chronic PTSD. Harv. Rev. Psychiatry.

[65] Slavich G.M., Irwin M.R. (2014). From stress to inflammation and major depressive disorder: A social signal transduction theory of depression. Psychol. Bull..

[66] Rosen R.L., Levy-Carrick N., Reibman J., Xu N., Shao Y., Liu M., Ferri L., Kazeros A., Caplan-Shaw C.E., Pradhan D.R. (2017). Elevated C-reactive protein and posttraumatic stress pathology among survivors of the 9/11 World Trade Center attacks. J. Psychiatr. Res..

[67] Haroon E., Raison C.L., Miller A.H. (2012). Psychoneuroimmunology meets neuropsychopharmacology: Translational implications of the impact of inflammation on behavior. Neuropsychopharmacology.

[68] Michopoulos V., Jovanovic T. (2015). Chronic inflammation: A new therapeutic target for post-traumatic stress disorder?. Lancet Psychiatry.

[69] Boscarino J.A. (1996). Posttraumatic stress disorder, exposure to combat, and lower plasma cortisol among Vietnam veterans: Findings and clinical implications. J. Consult. Clin. Psychol..

[70] Heim C., Ehlert U., Hellhammer D.H. (2000). The potential role of hypocortisolism in the pathophysiology of stress-related bodily disorders. Psychoneuroendocrinology.

[71] Maes M., Lin A.H., Delmeire L., Van Gastel A., Kenis G., De Jongh R., Bosmans E. (1999). Elevated serum interleukin-6 (IL-6) and IL-6 receptor concentrations in posttraumatic stress disorder following accidental man-made traumatic events. Biol. Psychiatry.

[72] Szotowski B., Antoniak S., Poller W., Schultheiss H.P., Rauch U. (2005). Procoagulant soluble tissue factor is released from endothelial cells in response to inflammatory cytokines. Circ. Res..

[73] von Känel R., Hepp U., Kraemer B., Traber R., Keel M., Mica L., Schnyder U. (2007). Evidence for low-grade systemic proinflammatory activity in patients with posttraumatic stress disorder. J. Psychiatr. Res..

[74] Kubzansky L.D., Koenen K.C., Spiro A., Vokonas P.S., Sparrow D. (2007). Prospective study of posttraumatic stress disorder symptoms and coronary heart disease in the Normative Aging Study. Arch. Gen. Psychiatry.

[75] Von Känel R., Dimsdale J.E., Patterson T.L., Grant I. (2003). Association of negative life event stress with coagulation activity in elderly Alzheimer caregivers. Psychosom. Med..

[76] Robicsek O., Makhoul B., Klein E., Brenner B., Sarig G. (2011). Hypercoagulation in chronic post-traumatic stress disorder. Isr. Med. Assoc. J..

[77] Austin A.W., Wirtz P.H., Patterson S.M., Stutz M., von Känel R. (2012). Stress-induced alterations in coagulation: Assessment of a new hemoconcentration correction technique. Psychosom. Med..

[78] Von Känel R., Kudielka B.M., Haeberli A., Stutz M., Fischer J.E., Patterson S.M. (2009). Prothrombotic changes with acute psychological stress: Combined effect of hemoconcentration and genuine coagulation activation. Thromb. Res..

[79] Von Känel R., Hepp U., Buddeberg C., Keel M., Mica L., Aschbacher K., Schnyder U. (2006). Altered blood coagulation in patients with posttraumatic stress disorder. Psychosom. Med..

[80] Austin A.W., Wissmann T., von Känel R. (2013). Stress and hemostasis: An update. Semin. Thromb. Hemost..

[81] Theofilis P., Sagris M., Oikonomou E., Antonopoulos A.S., Siasos G., Tsioufis C., Tousoulis D. (2021). Inflammatory Mechanisms Contributing to Endothelial Dysfunction. Biomedicines.

[82] Christiansen D.M., Berke E.T. (2020). Gender- and sex-based contributors to sex differences in PTSD. Curr. Psychiatry Rep..

[83] Breslau N. (2002). Gender differences in trauma and posttraumatic stress disorder. J. Gend. Specif. Med..

[84] Zlotnick C., Zimmerman M., Wolfsdorf B.A., Mattia J.I. (2001). Gender differences in patients with posttraumatic stress disorder in a general psychiatric practice. Am. J. Psychiatry.

[85] Cohen H., Matar M.A., Joseph Z. (2013). Animal models of post-traumatic stress disorder. Curr. Protoc. Neurosci..

